# The Inebriates Acts, 1897-1900

**Published:** 1907-11-09

**Authors:** 


### HOSPITAL ADMINISTRATION.

### CONSTRUCTION AND ECONOMICS.

### THE INEBRIATES ACTS, 1897-1900.

The Report of Dr. R. Welsh Branthwaite, the Inspector under the Inebriates Acts, for the year 1896, has recently been published as a Blue Book by the Home Office, and it contains much valuable information concerning the work of the certified Inebriate Reformatories which have been established under the Inebriate Acts, 1897-1900.

In addition to a mass of statistics and particulars respecting the numbers of persons sentenced to detention and admitted into reformatories during the past year Dr. Branthwaite deals very fully with the mental characteristics, general conduct, and moral character of persons thus committed. He points out the great need for a more careful classification of the inmates of reformatories, and the economic advantages of this method of dealing with inebriates.

After seven years' experience of the working of the Inebriates Act, Dr. Branthwaite is emphatically of opinion that the old method of sentencing hopelessly drunken persons to a term of imprisonment is entirely useless and wasteful. For the individual it only causes suffering, misery, and progressive mental, moral, and physical degradation; while on the other hand it does nothing for the community towards the prevention of disorder, danger to others, and useless expenditure of public funds.

There has been little or no sign of improvement in recent years in the type and general character of those persons who have been sent to reformatories for cure and treatment, the same large proportion being mentally defective, hopelessly irreformable, and fit for little else than permanent detention.

The Report shows that only 37 per cent. of the persons committed to reformatories are of average mental capacity, either when admitted or after six months' detention. While the remaining 63 per cent. are either: (1) Insane, certified and sent to asylums; (2) Very Defective, such as imbeciles, epileptics, etc.; or (3) Defective, eccentric, silly, or senile, or subject to periodical paroxysms of ungovernable temper.

The larger proportion of these persons are the victims of bad heredity, the number of those who become habitual drunkards from an occasional excessive indulgence in alcoholic liquors being comparatively small.

Concerning the general conduct of inmates of reformatories, it is stated that fully 56 per cent. of all cases admitted are well behaved—a surprisingly large proportion taking into consideration the degraded condition to which these persons have fallen and their troublesome conduct before committal. Apart from drunkenness, the behaviour of the larger majority has been contrary to the moral code which regulates the conduct of members of a civilised community. Not more than 15 per cent. of the women thus committed have led decent domestic lives up to the time of their admission to the reformatory, and the proportion of men is still smaller; the association of drunkards and immorality is proverbial, but very few persons realise the true significance of the combination.

Briefly stated, the accumulated experience points to three definite conclusions regarding the control and treatment of inebriates:—

1. That all habitual drunkards, who render themselves liable to be dealt with under the Inebriates Act of 1898, have become temporarily or permanently diseased and have passed beyond the limits of responsibility.

2. That any attempt to frighten such persons into sobriety by repeated fines or imprisonment has proved utterly useless.

3. That some more satisfactory method is urgently needed to afford the victims a chance of recovery, and, if recovery is past hope, then to safeguard society from malicious, criminal, or indecent results of his drunken habits.

The only possible remedy, therefore, appears to be to substitute long-continued reformatory treatment for the prison methods which still so largely obtain interest.

Much valuable information is furnished in the report as to the initial cost of some of the more recently erected reformatories. Dr. Branthwaite furnishes the following statistics respecting the cost of erection and equipment of three classes of institutions, namely—

| | Number of Inmates. | Cost per Bed. £ |
|---|---|---|
| Reformatories established by Local Authorities ... ... ... ... | 378 | 526 |
| Reformatories established jointly by Local Authorities and other persons | 240 | 200 |
| Reformatories established by voluntary bodies or private persons ... | 649 | 112 |

Full credit is given to the National Institution for Inebriates for leading the way in the direction of inexpensive accommodation, and the following particulars, together with the plans of three of their reformatories, are furnished in the report:—

| | Number of Inmates. | Cost per Bed. £ s. d. |
|---|---|---|
| Eastern Counties Inebriate Reformatory at East Harling, Norfolk | 300 | 99 18 8 |
| Southern Counties Inebriate Reformatory at Lewes, Sussex ... | 150 | 107 15 2 |
| North Midland at Ackworth, Yorkshire ... ... ... ... | 90 | 100 16 2 |
| Ditto (proposed extension)... | 250 | 96 19 2 |
| Totals ... ... ... | 790 | 100 11 8 |

It is evident from these statistics that the institutions which are provided by voluntary efforts have been erected at much less cost than those paid for out of public funds.

When we compare the above figures with the cost of some of the more recently erected sanatoria for consumptive patients, it is difficult to resist the conviction that a great deal of money had been unnecessarily expended upon the erection of some of these institutions, especially where it is borne in mind that their work and methods are still very largely of an experimental character, and may have to undergo considerable modifications in the near future.

We quite realise that there is no ground for comparison of the cost of Inebriate Reformatories with that of general hospitals, as the conditions of their work are so entirely different. At the same time, there can be no doubt that if arrangements could be made for the erection of a model hospital in the country on the lines advocated by Sir Henry Burdett in an article in The Hospital of December 2, 1905, we should find that the initial cost of hospital buildings could be greatly reduced, while the cost of administration and working such an institution would be reduced to a minimum.

Dr. Branthwaite's report is a most valuable contribution to the difficult problem of how best to deal with habitual drunkards, and contains much general information which deserves the most careful consideration.

